# ADHD diagnosis and methylphenidate consumption in children and adolescents: A systematic analysis of health databases in France over the period 2010–2019

**DOI:** 10.3389/fpsyt.2022.957242

**Published:** 2022-10-10

**Authors:** Sébastien Ponnou, Benoît Thomé

**Affiliations:** ^1^CIRNEF (EA 7454), University of Rouen Normandy, Mont-Saint-Aignan, France; ^2^Median Conseil, Pau, France

**Keywords:** ADHD, methylphenidate, children, adolescents, diagnosis, prevalence, off-label prescriptions, scholar and social determination

## Abstract

**Context:**

ADHD is the most common mental disorder in school-aged children. In France, methylphenidate is the only drug authorized for ADHD. Here, we describe the pattern of ADHD diagnosis and methylphenidate prescription to children and adolescents from 2010 to 2019.

**Methods:**

We conducted a retrospective cohort study of all beneficiaries of the French general health insurance scheme (87% of the population, 58 million people). We extracted information for all children and adolescents aged 0–17 years who received: (1) A diagnosis of ADHD (34,153 patients). (2) At least one methylphenidate prescription (144,509 patients). We analyzed the clinical, demographic, institutional, and social parameters associated with ADHD diagnosis and methylphenidate consumption in France.

**Results:**

The ADHD diagnosis among children and adolescents increased by 96% between 2010 and 2019. ADHD diagnosis affects more boys than girls. About 50.6% of children hospitalized with a diagnosis of ADHD in 2017 also had another psychiatric diagnosis. The rate of children hospitalized with an ADHD diagnosis and treated with MPH varied between 56.4 and 60.1%. The median duration of MPH treatment for a 6-year-old ADHD child initiated in 2011 is 7.1 years. In 2018, 62% of ADHD children were receiving at least one psychotropic medication. Between 2010 and 2019, methylphenidate prescription increased by +56% for incidence and +116% for prevalence. The prevalence of methylphenidate prescription reached between 0.61 and 0.75% in 2019. Boys are predominantly medicated. The median duration of treatment among 6-year-olds in 2011 was 5.5 years. The youngest children received the longest treatment duration. Diagnoses associated with methylphenidate prescription did not always correspond to the marketing authorization. Among children receiving the first prescription of methylphenidate, 22.8% also received one or more other psychotropic drugs during the same year. A quarter of initiations and half of renewals were made outside governmental recommendations. Educational and psychotherapeutic follow-up decreased from 4.1% in 2010 to 0.8% in 2019. French children and adolescents, who were the youngest in their class were more likely to be diagnosed (55%) and prescribed methylphenidate (54%). Children from disadvantaged families had an increased risk of ADHD diagnosis (41.4% in 2019) and methylphenidate medication (25.7% in 2019).

## Introduction

Attention Deficit Hyperactivity Disorder (ADHD) is considered the most common mental disorder in school-aged children (1). For this reason, it has been the object of thousands of studies worldwide.

Despite decades of intensive research, there are currently no neurological markers, genetic markers, or biological tests to identify or confirm the diagnosis of hyperactivity (1–8).

Thus, the description of ADHD relies exclusively on evaluating behavioral symptoms, namely, an attention deficit with or without motor impulsivity and hyperactivity. For this reason, the prevalence of ADHD is intensely debated at the international level, with significant variations depending on countries, regions, and the survey methods used. A systematic review of more than 150 prevalence studies shows significant variations ranging from 0.4 to 16.6% of school-aged children (9, 10). Beyond the demographic or cultural differences of the countries or regions surveyed (Europe, the Americas—including the United States—and Asia), the analyses show that the prevalence rates of hyperactivity are determined by the research method used, including clinical studies, telephone surveys, and questionnaires given to parents and/or teachers (10). Unfortunately, these different types of methods present numerous biases that question or even invalidate their significance: variations related to diagnostic criteria, scales and analysis grids, sampling, level of training of the interviewers, and level of information of the respondents, to the taking into account of the risks of co-morbidity and to diagnostic errors or social factors likely to influence the diagnosis (11–22).

Similarly, treatment recommendations vary considerably between countries (1). In North America, drug treatment is recommended as the first line of treatment, whereas in most European countries, a psychotherapeutic, educational, and social approach is preferred. Although medication should be reserved for the most severe cases, it is used early (1). In 2012, among the ADHD medications available, methylphenidate (MPH) was by far the most commonly prescribed in European countries. In the United States, MPH accounts for only half of the prescriptions and amphetamine-based drugs account for 35% (23). Atomoxetine represents an alternative to these psychostimulants, but its prescription rate remains low: it is highest in Denmark (18% in 2012) (23). In all Western countries, the prescription of medication for ADHD increased rapidly between 1990 and 2010. It then stabilized in some countries (e.g., the United Kingdom and Denmark) while the increase continued, albeit more slowly, in other countries (e.g., the United States and Iceland) (23, 24). However, the prescription rate during the period 2012 to 2015 differs considerably from country to country: it is around 5% in the United States and Iceland, but 0.5% in the United Kingdom (23, 24). Although the prescription of psychostimulants may provide short-term relief from the behavioral symptoms of ADHD and facilitate the child's care and education, several epidemiological studies that have followed very large cohorts over many years show that stimulants have no long-term benefit on the risks of academic difficulties, delinquency, and substance abuse associated with ADHD (25–27). Evidence is mounting that ADHD medications does not make a difference to schoolwork or achievement (28).

Otherwise, numerous studies have shown the influence of the school system on the diagnosis and medication of children with ADHD. For example, in one city in the state of Virginia, 63% of school children who were 1 year ahead of their peers were treated with psychostimulants (29). In the general American population, the prevalence of ADHD varies relative to the month of birth, confirming that the youngest schoolchildren in their class are more frequently diagnosed (30, 31). A Canadian study showed that the number of boys treated with a psychostimulant is 41% higher if they were born in December than if they were born in January. For girls, the rate is 77% (32). Elsewhere, Elder (30) shows that the hyperactive behavior of the youngest children in a class is more frequently judged pathological by their teachers than by their parents. American teachers are pressured by their superiors to report possible cases of ADHD to parents. Indeed, since the passing of a 1990 law, American schools have received an additional allocation, which varies according to the county, for each child diagnosed, and the pharmaceutical industry now provides teachers with the documentation necessary to identify potential cases (33). Finally, schools are evaluated according to the performance of their students and are therefore encouraged to raise their academic level. A study comparing American states positively correlated the binding nature of these incentives with the prevalence of ADHD (34). These surveys have been duplicated internationally, with the same phenomenon identified in Norway, Lombardy (Italy), Finland, and the United Kingdom. Beyond the differences in the educational systems of these countries, the meta-analyses currently available support the hypothesis of the influence of the school system on the diagnosis of ADHD and on the medication of children (35, 36).

Similarly, many environmental and social risk factors have been identified. These include exposure to toxic levels of lead (37), premature birth (38, 39), severe child abuse, parents with mental disorders, poor interactions between parents and children (40–43), low academic or economic level of the parents, and being part of a single-parent family or born of a teenage mother (42, 44). Excessive exposure to television before the age of 3 years also seems to be particularly harmful to the development of a child's attention span (45–47).

Unfortunately, this type of study is lacking in France, meaning that questions concerning the prevalence of ADHD, drug prescription, and the influence of academic and social factors on diagnosis and prescription are still the object of much controversy.

Indeed, the only prevalence study currently available in France was financed by the drug industry. It reports a high prevalence of ADHD (3.5–5.6% of children) based on a telephone survey entrusted to a polling institute and carried out by non-specialist operators trained on the fly (48). This study is currently a reference in terms of policies and care practices dedicated to ADHD children in France.

In terms of medication, the only compound authorized in France for the treatment of ADHD is MPH, first commercialized in 1995. It is marketed in a simple form (Ritalin^®^) and a delayed form (Ritalin-LP^®^, Concerta^®^, Quasym^®^, Medikinet^®^). MPH is recommended for children aged 6 years and over “when psychological, educational, social, and family corrective measures alone are insufficient” (49). Prescription is subject to strict limitations and conditions of delivery: initial prescription and annual renewals carried out in a hospital setting by specialists (until September 2021), monthly renewals following specific prescription protocols, and the identification of the pharmacist filling the prescription (49). Data describing MPH prescription in children and adolescents in France have been published in five peer-reviewed journals (50–54). The populations studied correspond either to a sample of about 1% of the French population of children covered by the general health insurance scheme (53), or to all of these children, but in only one region (50) or two regions (51, 54), or finally to all of the children covered by the social regime for the self-employed (4.5% of the French population) (52). Two studies describe the evolution of the prevalence of MPH prescription over several years (2003–2005 and 2005–2011, respectively) (51, 52). The other three do not report annual changes in this prevalence, and the most recent data are for the period from 1 July 2010 to 30 June 2013 (54). By far the most comprehensive and recent data are available in a 2017 report by the French National Security Drug Agency (49). These data concern all children insured under the general health insurance scheme and describe the evolution of MPH prescriptions between 2008 and 2014.

There are currently no data concerning the influence of the school system or social risk factors on the diagnosis or medication of ADHD in France.

Given this context, epidemiological studies and the analysis of health databases constitute a tool for producing new data concerning the diagnosis and care of children with ADHD (55, 56). For this special issue of *Frontiers in psychiatry* on the theme of “ADHD: science and society,” we examined the medico-administrative databases in France, particularly:

- ADHD diagnosis in France among children and adolescents with a medical-surgical-obstetric (MCO) or Psychiatric (PSY) hospitalization between 2010 and 2019 (excludes day hospitalizations in psychiatry);
- The pattern of MPH prescription to children in France in the same period;
- The influence of the school system and social inequalities on ADHD diagnosis and psychostimulant prescription.

This article presents unpublished data on the evolution of ADHD diagnosis in France and represents by far the most comprehensive and robust analysis of the multiple clinical, demographic, institutional, and social variables concerning MPH prescription in that country.^1^

## Methods

### Population

We conducted a retrospective cohort study of all beneficiaries of the general health insurance scheme, including local mutual health insurance companies, i.e., nearly 87% of the French population, more than 58 million people.^2^ Our cohort does not take into account patients covered by the social regime for the self-employed or by the farmers' and farmworkers' social mutual fund. This database lists the different health insurance schemes in France.

We extracted information for all children and adolescents aged 0–17 years who received:

1) A diagnosis of ADHD/hyperkinetic disorder during an MCO or PSY hospitalization (excludes day hospitalizations in psychiatry) between 2010 and 2019 (F.90 and subtypes);^3^ and/or
2) At least one MPH prescription during the period 2010–2019. For each patient, the date of inclusion in the cohort was defined as the date of the initial delivery of MPH [ATC class N06BA04 (*Anatomical Therapeutic Chemical Classification System*)].

Two cohorts were thus established. The first cohort was composed of 38,183 children and adolescents hospitalized with an ADHD diagnosis between 2010 and 2019 in France in MCO or PSY facilities. From these, we excluded 3,920 patients who were not listed properly in the French National Health Data System databases (e.g., children appearing under two numbers corresponding to each of their parents). Thus, we selected a final cohort of 34,153 children or adolescents aged 17 years or younger with a diagnosis of ADHD between 1 January 2010 and 31 December 2019. Because diagnostic coding is mandatory in France only in the case of full-time hospitalization, the cohort is not necessarily fully representative and can provide only limited information. It may also be the case that this cohort brings together the children with the most severe symptoms of ADHD, since these children were hospitalized as inpatients for ADHD in psychiatry.

**Figure 1:**
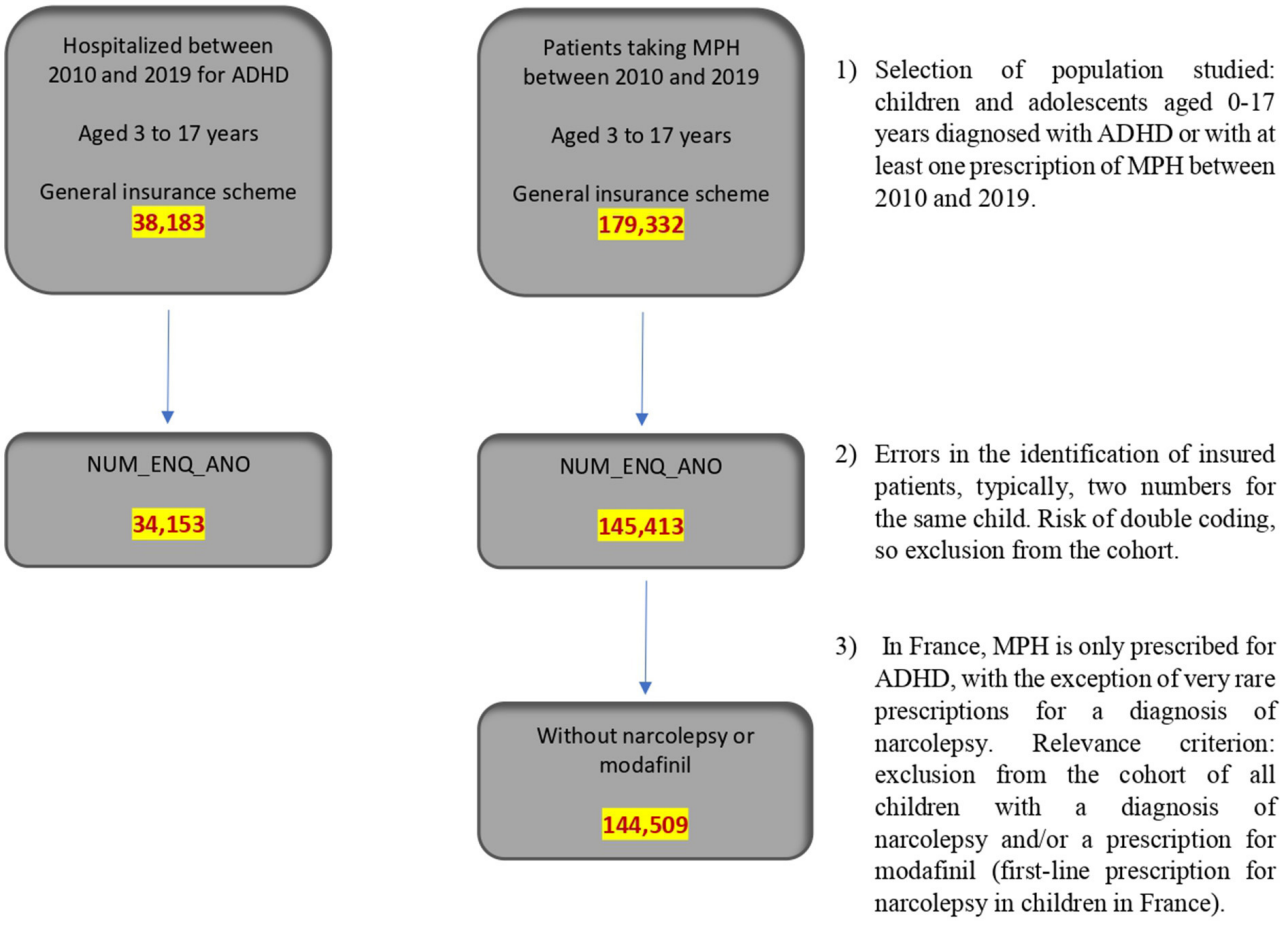
Population studied.

The second cohort included 179,332 children and adolescents who received at least one prescription of MPH between 2010 and 2019. From these, we excluded 33,919 patients who were not listed properly in the French National Health Data System databases. We also excluded 904 patients whose MPH prescription was associated with narcolepsy identified either by explicit diagnosis or by co-prescription of modafinil. For this cohort, we therefore retained 144,509 children or adolescents up to 17 years of age who were identified with at least one prescription of MPH between 1 January 2010 and 31 December 2019 (Figure 1).

Each of the two cohorts was treated and analyzed separately.

### Data collection

#### ADHD diagnosis in children hospitalized in France

For each patient in our ADHD cohort, we used the following information:

- Date of birth and sex;
- The date of diagnosis;
- Possible co-diagnoses or co-morbidities. We obtained these co-diagnoses from the French national health insurance office (*Caisse Nationale de l'Assurance Maladie* or CNAM), which has established a so-called mapping. Indeed, the CNAM makes use of algorithms defining 56 groups of pathologies, taking into account diagnoses in hospitals, psychiatric institutions, and follow-up and rehabilitation care, as well as registration on the list of long-term conditions for the current year and drugs consumed during the past years. We made use of co-morbidities and co-diagnoses for ADHD patients in 2017, the most recent year for which we had CNAM mapping data. To distinguish between certain childhood psychiatric disorders and ADHD, we reconstructed the CNAM algorithm taking into account hospitalizations for MCO, PSY, and long-term conditions.
- If relevant, the prescription of MPH over the period covering 2010–2019;
- If relevant, the prescription of psychotropic medications, that is, all ATC classes N03, N04, N05, and N06, but only within 12 months after hospitalization;
- Durations of treatment.

#### MPH consumption pattern in France between 2010 and 2019

For each patient in our MPH cohort, we collected the following information:

- Date of birth and sex;
- The date of the initial prescription in the period studied (2010–2019);
- The date of the end of treatment. We selected the date that met the first valid criterion among the following three possibilities: (1) the date of the last MPH delivery between 2010 and 2019 +30 days (the delivery of MPH in France is packaged for a 30-day consumption); (2) the date on which the patient reached the age of 18; and (3) the end date of the study, i.e., 31 December 2019;
- The number of deliveries of MPH prescribed per patient;
- When possible, co-morbidities are associated with the prescription of MPH. Recall that it is possible to obtain a co-morbidities from the French health. In total, regarding co-morbidities, the most recent CNAM data are for children whose treatment was initiated in 2017, and the CNAM recorded a diagnosis for only 28.6% of them. Because it is not known whether this subpopulation is representative of the whole, co-morbidities associated with MPH prescription should be viewed with caution:
- If relevant, the prescription of psychotropic medications in addition to MPH;
- The place (e.g., hospital) where the initial prescription was delivered as well as the type of physician (e.g., general practitioner) who prescribed it;
- If relevant, any visits to a French medical-psychological-pedagogical center (*Center Médico-Psycho-Pédagogique*, or CMPP).

#### The influence of the school system and social inequalities on ADHD diagnosis and the prescription of psychostimulants to children and adolescents in France

With regard to the influence of the school system on ADHD diagnosis and MPH prescription, we were interested in the distribution of diagnoses and psychostimulant consumption according to the child's age based on the month of birth. We focused on this variable because it is the main discriminating factor put forward in the international literature (36).

Regarding the impact of social factors on ADHD diagnosis and MPH prescription, we used two main criteria:

- Among the children in our two cohorts, we looked for those whose families benefited from the French universal health coverage [*Couverture Maladie Universelle*, or CMU, since 2016 called Universal health protection (*Protection Universelle MAladie or PUMA*)] or complementary health insurance (CMU-C), or complementary health insurance payment aid (*Aide au paiement d'une Complémentaire Santé*, or ACS).^4^
- We also looked for children coded in the hospital databases as having unfavorable social conditions (ICD-10 codes Z55, Z59, Z61–Z64^5^) among the patients in our two cohorts.

The entire research protocol was validated by the French ethics and science committee for research, studies, and evaluations in the field of health (CESREES),^6^ as well as the French National Commission for Information Technology and Civil Liberties.^7^ The research was part of an agreement with the CNAM.

### Data analysis

The patients at the time of hospitalization/diagnosis or prescription were divided into four categories, built from the recommendations of the ANSM (49) and the European Medicines Agency: 0–2, 3–5, 6–11, and 12–17 year.^8^ Patients were assigned to these categories according to the lower of the two ages they were during the year under consideration.

For the MPH cohort, the annual incidence, i.e., the number of patients receiving an initial MPH prescription over the period studied, was not calculated for 2010, because a prescription in 2010 could have been preceded by a diagnosis or prescription in 2009 that would not have been taken into account in our study. From 2011 onwards, an initial prescription date implied an absence of the prescription for at least 12 months. This seemed sufficient to consider it a true initial prescription.

For the MPH cohort, the annual prevalence, i.e., the number of children and adolescents receiving a prescription for MPH in the year under consideration, was calculated as a percentage of the general population in the same age-group, using annual data from the French National Institute of Statistics and Economic Studies (*Institut national de la statistique et des études économiques*, or INSEE). This percentage was adjusted to take into account the fact that our cohort included only children insured by the general health insurance scheme and therefore did not take into account 13% of the population. However, we did not take into account the exclusion of patients with problematic numerical identification. Although we can say with certainty that the number of excluded patients is < 33,919, it was not possible to count them accurately. The prevalence presented here therefore underestimates the actual prevalence. This prevalence could be obtained only by multiplying the observed prevalence by a coefficient, which is impossible to identify precisely, ranging from 1 to 1.23.

Given the limitations presented for the ADHD cohort, it did not seem relevant to establish a diagnostic rate or an incidence/prevalence rate.

For the two cohorts, the duration of MPH consumption was calculated for each patient in terms of days as being the period between the date of initial prescription and the date of the end of treatment. We did not take into account times when treatment was interrupted and later resumed. This duration of treatment was only calculated for patients who started their treatment in 2011, as it enables to have the longest observation. For the years closest to the end of our study (2019), the absence of data concerning the possible extension of treatment beyond 31 December 2019 would have led to an increasingly significant underestimation of the actual duration of treatment.

### Limitations

The MPH cohort records all prescriptions of this compound to all child patients in France over 10 years. For this reason, this cohort has given rise to an intensive study.

However, the data concerning the ADHD cohort must be considered with caution since, as we have pointed out, diagnostic coding is only mandatory in France in the case of inpatient hospitalization. Outpatient hospitalizations or out-of-hospital consultations do not give rise to diagnostic coding in health databases. Therefore, the ADHD cohort included all children and adolescents hospitalized with an ADHD diagnosis between 2010 and 2019. For this reason, the analysis of this cohort gives us only partial information on the evolution and the different variables likely to contribute to ADHD diagnosis in France. It cannot be used to establish a diagnostic rate or a prevalence rate. The only means to obtain these rates would be through joint access to the databases of the French social security system and of the French departmental disability center (*Maison Départementale des Personnes Handicapées*). Such access will not be possible until 2023. Given the relative representativeness of this cohort, we limited our research to the following main criteria, which seemed to provide both novel and robust scientific information on ADHD diagnosis in hospitalized children in France: the evolution of the diagnosis between 2010 and 2019, co-morbidities, or co-diagnoses associated with ADHD diagnosis, the rate of children diagnosed with ADHD and treated with MPH, prescriptions of psychotropic drugs to hospitalized ADHD children, and the influence of school and social factors on the diagnosis. As mentioned above, it may be the case that this cohort includes those children with the most severe symptoms of ADHD.^9^

## Results

### Evolution of ADHD diagnosis in France between 2010 and 2019

#### ADHD diagnosis among children hospitalized full-time in France between 2010 and 2019

The ADHD diagnosis among children and adolescents in France increased steadily by 96% between 2011 and 2019 (Table 1). Diagnoses in 0–2-year-olds were very low between 2010 and 2019 and rose little among 3–5-year-olds. In contrast, the number of children diagnosed with ADHD and hospitalized in France increased by 167% among 12–17-year-olds.

**Table 1:** Trends in the number of patients coded as ADHD in health databases by age-group and year.

	**0–2 years**	**3–5 years**	**6–11 years**	**12–17**	**Total**
2010	9	437	2,018	717	3,181
2011	11	459	2,278	854	3,602
2012	5	454	2,628	1,013	4,100
2013	1	380	2,382	1,017	3,780
2014	9	393	2,410	1,138	3,950
2015	12	472	3,146	1,326	4,956
2016	11	501	3,359	1,502	5,373
2017	10	451	3,326	1,456	5,243
2018	2	553	3,655	1,747	5,957
2019	0	435	3,888	1,915	6,238

ADHD diagnosis affects more boys than girls. This proportion remained relatively stable between 2010 and 2019: 81% boys−19% girls in 2010; 79% boys−21% girls in 2015; 77% boys−23% girls in 2019.

#### Diagnoses and co-morbidities associated with ADHD among hospitalized children

We examined the psychiatric diagnoses and co-morbidities associated with ADHD for incident inpatients in 2017, the most recent year for which we had CNAM mapping data. We found that 50.6% of children hospitalized with ADHD in 2017 also had another psychiatric diagnosis coded in the health databases: mood disorders (6.7%), mental disabilities (3.3%), psychotic disorders (0.7%), and various other child psychiatric disorders (6.1%, for example, autism and various learning disorders) and addictions (0.5%).

However, these co-morbidities and diagnoses associated with ADHD are not evenly distributed across the ages of the children. They concern:

- 52.4% of 3–5-year-olds, with childhood disorders other than ADHD (48.1%), mental disability (5.5%), autism and other learning disorders (4.2%), neurotic and mood disorders (0.7%), and psychotic disorders (0.5%).
- 49% of 6–11-year-olds, with childhood disorders other than ADHD (42.9%), neurotic and mood disorders (3.5%), mental deficiency (3.1%), autism and other learning disorders (4.8%), and psychotic disorders (0.2%).
- 54.1% among 12–17-year-olds, with childhood disorders other than ADHD (36.6%), neurotic and mood disorders (17.9%), autism and other learning disorders (10.2%), mental deficiency (2.8%), psychotic disorders (2.2%), and addictive disorders (1.9%).

Some children have multiple co-morbidities and diagnoses associated with ADHD.

#### MPH prescription rates among children hospitalized with an ADHD diagnosis

We also examined the percentage of children hospitalized with an ADHD diagnosis and treated with MPH. Between 2011 and 2017, the ratio of children diagnosed with ADHD and having never taken MPH ranged from 39.9 to 43.6% (Table 2). The data for 2018 and 2019 should be viewed with caution since we have limited hindsight for these 2 years. This accounts for lower medication rates.

**Table 2:** The ratio of children treated with MPH among children with ADHD hospitalized in France between 2011 and 2019.

	**ADHD**	**ADHD without MPH**	**%**
2011	2,806	1,207	43
2012	2,928	1,228	41.9
2013	2,730	1,134	41.5
2014	2,845	1,136	39.9
2015	3,663	1,530	41.8
2016	3,773	1,623	43
2017	3,854	1,681	43.6
2018	4,240	2,117	49.9
2019	4,241	2,328	54.9

In other words, the rate of children hospitalized with ADHD and treated with MPH in France varied between 56.4 and 60.1% (Table 2). This variation was relatively stable between 2011 and 2017.

We examined the median duration of MPH treatment for children hospitalized with ADHD in 2011. For a 6-year-old child who started treatment in 2011, the median duration of treatment was 2,580 days, that is, 7.1 years.

#### Co-prescription of psychotropic drugs

More generally, we examined all prescriptions of psychotropic drugs associated with ADHD diagnosis to hospitalized children between 2010 and 2018.^10^ In 2018, 62% of ADHD children were receiving at least one psychotropic medication: stimulants (45.9%), antipsychotics (24.2%), anxiolytics (10.9%), antidepressants (5.5%), antiepileptics (6.5%), or hypnotics (3.3%).^11^

The main drugs used the year fallowing an hospitalization in 2018 were MPH (Ritalin^®^, derivatives and/or generics−45.9%), risperidone (Risperdal^®^, derivatives and/or generics−14.9%), cyamemazine [Tercian^®^, hydroxyzine (Atarax^®^, derivatives and/or generics−9%), derivatives and/or generics−7.1%], aripiprazole (Abilify^®^, derivatives and/or generics−5.8%), valproic acid (Depakine^®^, derivatives and/or generics−2.7%), midazolam (Hypnovel^®^ derivatives and/or generics−2.6%), sertraline (Zoloft^®^, derivatives and/or generics−2.1%), Diazepam (Valium^®^, derivatives and/or generics−1.7%), and tropatepine (Lepticur^®^, derivatives and/or generics−1.7%).

Of 62% of ADHD children consuming medications in the year following their initial diagnosis/hospitalization, 60.5% received one treatment, 25.3% received two treatments, 10.1% received three psychotropic drugs, and 4% received four or more psychotropic medications.

### Analysis of the consumption pattern of MPH in children and adolescents in France

#### Evolution of MPH consumption in France between 2010 and 2019

The annual incidence of MPH prescriptions increased steadily between 2011 and 2019 (Table 3). All ages combined, this increase reached 56.7% over the period (10,065 incident patients in 2011, 15,776 in 2019). This increase was mainly in 6–11-year-olds (+62.9%) and 12–17-year-olds (+48.4%). In comparison, prescription for 3–5-year-olds was low and increased less (+20.8%). Lastly, prescription for 0–2-year-olds was rare. These increases cannot be explained by an increase in the general population, as the French population of under-20s decreased (−3.6%) between 2010 (15.97 million) and 2019 (15.39 million).

**Table 3:** Number of patients with an initial MPH prescription by age-group and year.

	**0–2 years**	**3–5 years**	**6–11 years**	**12–17**	**Total**
2011	7	495	6,792	2,771	10,065
2012	3	511	7,115	2,749	10,378
2013	5	558	7,458	2,780	10,801
2014	5	566	8,461	3,243	12,275
2015	0	546	9,133	3,557	13,236
2016	3	545	9,416	3,527	13,491
2017	1	595	9,905	3,802	14,303
2018	0	576	10,214	3,824	14,614
2019	1	598	11,064	4,113	15,776
Total	25	4,990	79,558	30,366	114,939

**Figure 2:**
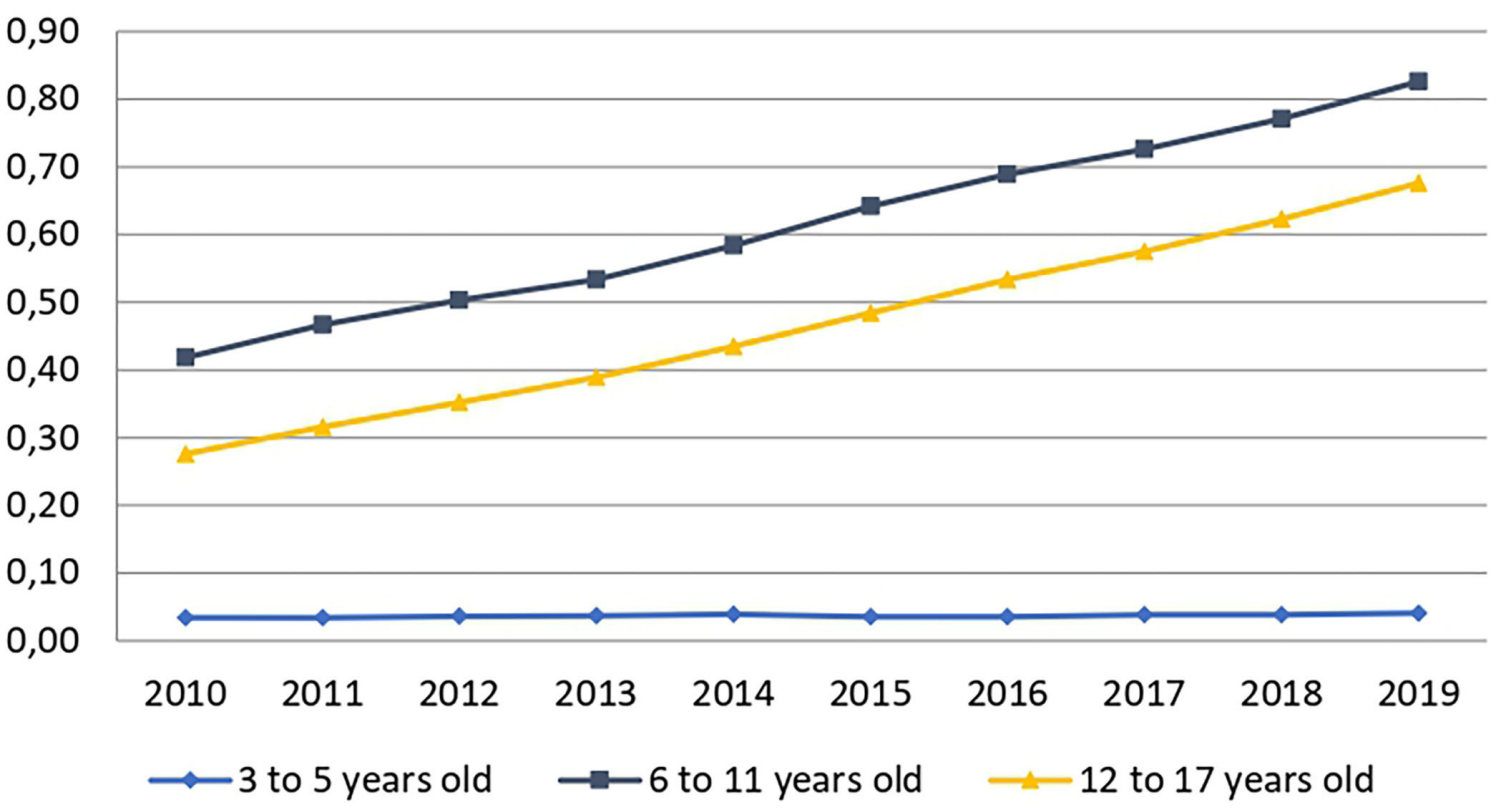
Changes in the prevalence rate of MPH prescription. This rate is expressed as a percentage of the general population for each age-group and time period.

Between 2010 and 2019, the prevalence of MPH prescription steadily increased (Figure 2). It nearly doubled for 6–11-year-olds (+98%) and increased even more for 12–17-year-olds (+145%). In contrast, among 3–5-year-olds, prevalence remained much lower and increased little (+21%).

The prescription of MPH concerned more than four boys to every girl, and this difference barely changed over the study period. Indeed, the percentage of boy patients was 82.5% in 2010 and 80.8% in 2019. Between 2010 and 2019, this percentage remained between 80.8% (in 2019) and 82.5% (in 2010).

#### Characteristics of MPH treatment

As explained above, the duration of treatment was calculated only for patients who received their initial prescription in 2011 (Table 4). The median durations observed for adolescents who started treatment at age of 16 or 17 years are certainly underestimated due to the fact that these patients exited the cohort on their 18th birthday. For the others, even if the longest durations for some patients were shortened by the cut-off date of their 18th birthday, this does not change the estimate of the median since its value in years is lower than the number of years between the patient's age at initiation of treatment and their 18th birthday. The number of 2- and 3-year-olds is too small to draw a firm conclusion about the duration of treatment.

**Table 4:** Median durations of MPH treatment for patients with an initial prescription in 2011.

**Age**	**Number of children**	**Median treatment times**
2	7	3,077 days (8.4 years)
3	44	1,227 days (3.4 years)
4	113	1,991 days (5.5 years)
5	338	1,870 days (5.1 years)
6	1,069	1,990 days (5.5 years)
7	1,352	1,581 days (4.3 years)
8	1,406	1,443 days (4 years)
9	1,359	1,254 days (3.4 years)
10	916	1,089 days (3 years)
11	875	757 days (2.1 years)
12	845	680 days (1.9 years)
13	669	552 days (1.5 years)
14	488	412.5 days (1.1 years)
15	346	279 days (0.8 years)
16	255	387 days (1.1 years)
17	168	248 days (0.7 years)

Thus, the median duration of MPH consumption among 6-year-old children in 2011 was 5.5 years and up to more than 8 years for 25% of them (Table 4). The table clearly shows that the younger the patient, the longer the duration of treatment (Table 4).

While the data collected do not allow us to give a precise idea of the evolution of the duration of treatment between 2011 and 2019, the comparison between prevalence and incidence sheds some light. Indeed, between 2010 and 2019, the prevalence of MPH prescription increased more than its incidence. This indication suggests that treatment durations increased between 2011 and 2019. To support this interpretation, we examined the MPH deliveries for each patient. Table 5 shows that the deliveries each year for each patient increased on average from 6.94 in 2010 to 7.95 in 2019 (Table 5). This increase is even more pronounced if only the number of deliveries during the 12 months after the initial prescription is counted (Table 6). This increase is similar regardless of the child's age-group (Table 6). These observations suggest that the number of children who discontinued treatment early decreased between 2011 and 2018.

**Table 5:** Average number of MPH deliveries per year per patient.

**Year**	**Total deliveries**	**Average number of deliveries per patient**
2010	183,696	6.94
2011	2,017,175	7.04
2012	233,801	7.21
2013	259,498	7.55
2014	290,653	7.62
2015	326,144	7.68
2016	360,736	7.79
2017	394,865	7.88
2018	424,823	7.98
2019	453,598	7.95

**Table 6:** Average number of MPH deliveries within 12 months of treatment initiation.

**Age-Group**	**2011**	**2012**	**2013**	**2014**	**2015**	**2016**	**2017**	**2018**
3–5 years	7.4	8.1	8.1	8.1	8.1	8.9	9.4	9.4
6–11 years	7.5	7.9	8.4	8.7	8.7	9.1	9.4	9.6
12–17 years	6.1	6.9	7.3	7.6	7.7	8.1	7.9	8.1

#### Diagnoses associated with MPH prescription

We were only able to examine the diagnosis associated with the MPH prescription for 3,965 children who started treatment in 2017. Of these, two-thirds (65.4%) were diagnosed with ADHD. In the remaining third, we found various psychiatric pathologies including mood disorder (8.4%), mental disabilities (7.7%), psychotic disorders (1%), and various other child psychiatric disorders (17.5%) (e.g., autism and various learning disorders). These very partial data must be considered with caution. However, they show that the recommendations of the French National Authority for Health (*Haute Autorité de Santé*, or HAS) are not always followed, given that according to the HAS, MPH prescription should be reserved for children diagnosed with ADHD.

#### Co-prescriptions of psychotropic drugs associated with MPH

We studied prescriptions for psychotropic medications during the 12 months following the initial MPH delivery in 2018. During this year, we observed that 22.8% of children were prescribed at least one other psychotropic medication in addition to MPH. These co-prescriptions belonged to various pharmacological classes: neuroleptics (64.5%), anxiolytics (35.5%), antidepressants (16.2%), antiepileptics (11%), hypnotics (4.8%), and antiparkinsonians (3%).

The main drugs prescribed were risperidone (Risperdal^®^, derivatives and/or generics−10.6%), hydroxyzine (Atarax^®^, derivatives and/or generics−6%), cyamemazine (Tercian^®^, derivatives and/or generics−3.9%) aripiprazole (Abilify^®^, derivatives and/or generics−2.7%), sertraline (Zoloft^®^, derivatives and/or generics−1.4%), valproic acid (Depakine^®^, derivatives and/or generics−1.1%), and fluoxetine (Prozac^®^, derivatives and/or generics−1%).

Of those children taking multiple psychotropic medications in the year following the initial MPH delivery, 63.5% received two treatments (MPH and another psychotropic drug), 20.8% received three psychotropic drugs, 8.5% received four, and 6.9% were prescribed five or more psychotropic drugs.

#### Prescriptions falling outside of official recommendations

**Figure 3:**
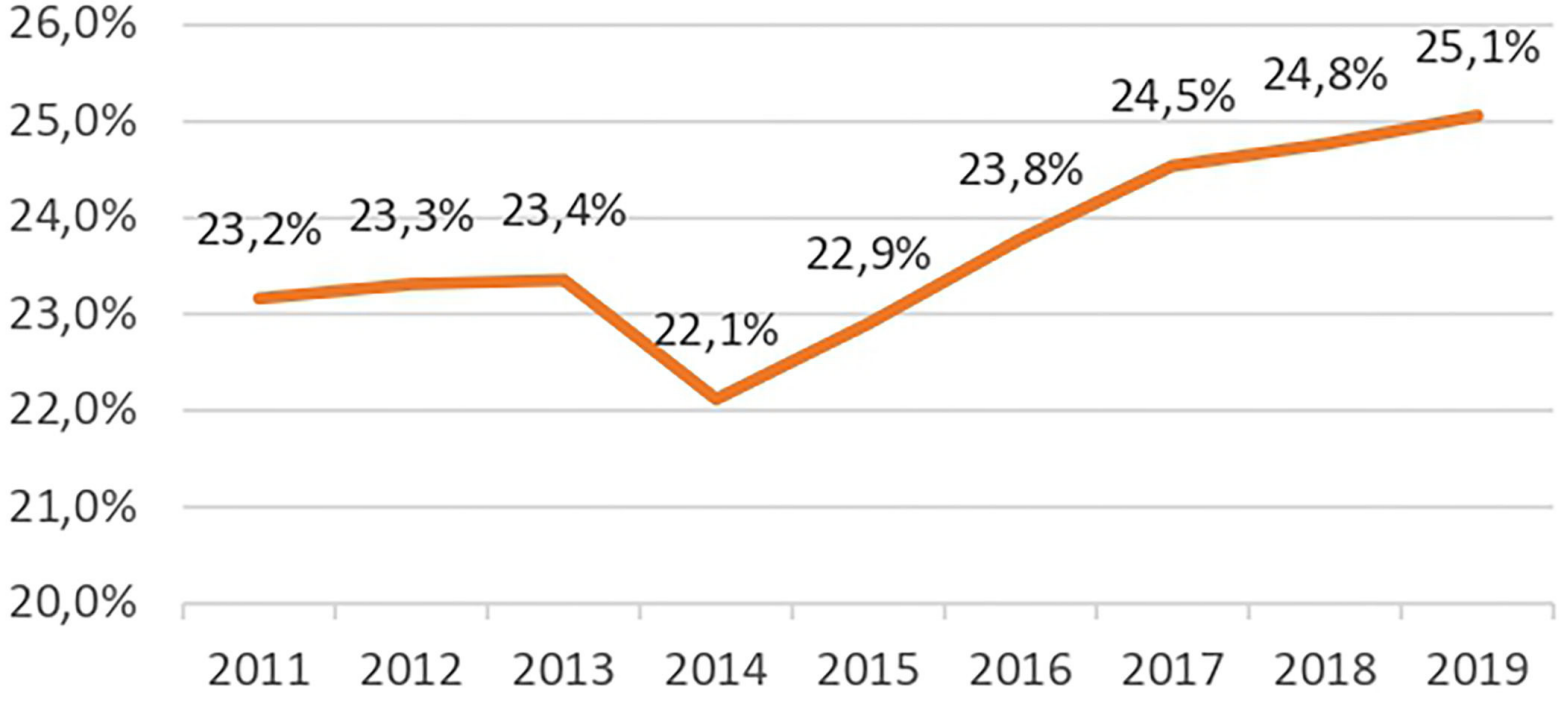
Evolution of initial out-of-hospital MPH prescriptions.

Until September 2021, an initial MPH prescription was required to be given in a hospital. Yet, during the period studied, nearly a quarter of initial MPH prescriptions were made in private practice or private MCO facilities. The reports and reminders of prescribing conditions carried out by the ANSM in 2013 and 2017 had only a limited effect on this practice (Figure 3).

In addition, French regulations required that the annual renewal of the MPH prescription be done during a specialized hospital consultation. To examine whether this requirement was met, we looked for the occurrence of a renewal of the MPH prescription in hospitals during the 14 months following the date of the initial prescription. For nearly half of the children (49.6%) having started treatment in 2015, 2016, and 2017, this was not the case. Even when accounting for the interruption of treatment within 1 year for some children, it appears that the annual on-site hospital refill requirement was not always met.

This observation suggests that an increasing number of general practitioners prescribe MPH in France. Thus, in 2010, 15,318 general practitioners wrote 97,819 prescriptions. The number of general practitioners who prescribed MPH almost doubled in 2019 (29,082), while the number of their prescriptions increased by 221% (314,801). Moreover, 87% of MPH prescriptions outside of hospitals were given by general practitioners. This proportion remained stable between 2010 and 2019.

However, it is noteworthy that nearly two-thirds of French general practitioners never prescribe MPH (29,082 prescribers out of 101,335 general practitioners in France in 2019). Among prescribing physicians, practices are very heterogeneous, with large variations in the number of prescriptions per physician in 2019 (mean 10.82; standard deviation 12.90; range 1–664). These results suggest that the majority of MPH prescriptions, as well as their increase between 2010 and 2019, were given by a minority of general practitioners.

Such heterogeneous practices can also be observed in hospitals: across the country, 358 of the 1,356 French public health establishments carried out 78.8% of hospital prescriptions^12^ in 2019. The top 30 establishments alone accounted for 28% of prescriptions.

#### Follow-up treatment in the prescribing hospitals and visits to medical-psychological-pedagogical centers

Insofar as MPH is not a first-line treatment for ADHD in France and can only be used once appropriate psychological, educational, social, and family measures have been taken (49), we were interested in the medical follow-up of children taking MPH in prescribing institutions during the 13 months following the beginning of treatment.^13^ Across all hospital services, the results show that the number of children receiving at least one medical consultation (all causes) with 13 months after the initial prescription ranged from 15.8 to 12.9% between 2011 and 2018. In other words, 84.2% to 87.1% of children treated did not receive medical follow-up by the hospital service that initiated treatment. In 2018, among the patients beginning treatment in a hospital, 632 children had one medical consultation (5.7%), 351 children had two consultations (3.2%), and 178 children had three consultations by the hospital service that initiated treatment (1.6%). These observations show that follow-up practices by the prescribing hospital services are far from being systematic.

We were also interested in the follow-up treatment of children in CMPPs. French CMPPs are the primary organizations providing psychosocial support for children and their families. The practices used in CMPPs represent, however, only a part of the care provided to children with ADHD. Although the raw values shown in Table 7 are difficult to interpret, their evolution between 2010 and 2019 seems interesting. Indeed, while MPH prescription steadily increased between 2010 and 2019, the number of visits to the CMPPs of children receiving a prescription of MPH steadily decreased over the period studied: between 2010 and 2019 it dropped by more than 75% (Table 7).

**Table 7:** CMPP visits among patients receiving a prescription for MPH.

**Year**	**Patients**	**Visits**	**Visits per patient**	**Total number of patients**	**Total %**
2010	1,305	21,083	16.16	31,453	4.1
2011	1,169	22,105	18.91	37,583	3.1
2012	1,244	23,963	19.26	42,282	2.9
2013	1,277	26,274	20.57	46,261	2.8
2014	1,292	27,171	21.03	51,041	2.5
2015	1,258	25,362	20.16	56,938	2.2
2016	1,301	26,073	20.04	62,028	2.1
2017	1,308	29,213	22.33	66,461	2.0
2018	817	15,769	19.30	70,103	1.2
2019	550	10,175	18.50	72,798	0.8

### Influence of the school system and social inequalities on ADHD diagnosis and the prescription of psychostimulants in children and adolescents in France

#### The influence of school on ADHD diagnosis

The diagnosis of hyperactivity is systematically correlated with the child's month of birth, meaning that the youngest students in a class are most at risk of being diagnosed as hyperactive.

**Figure 4:**
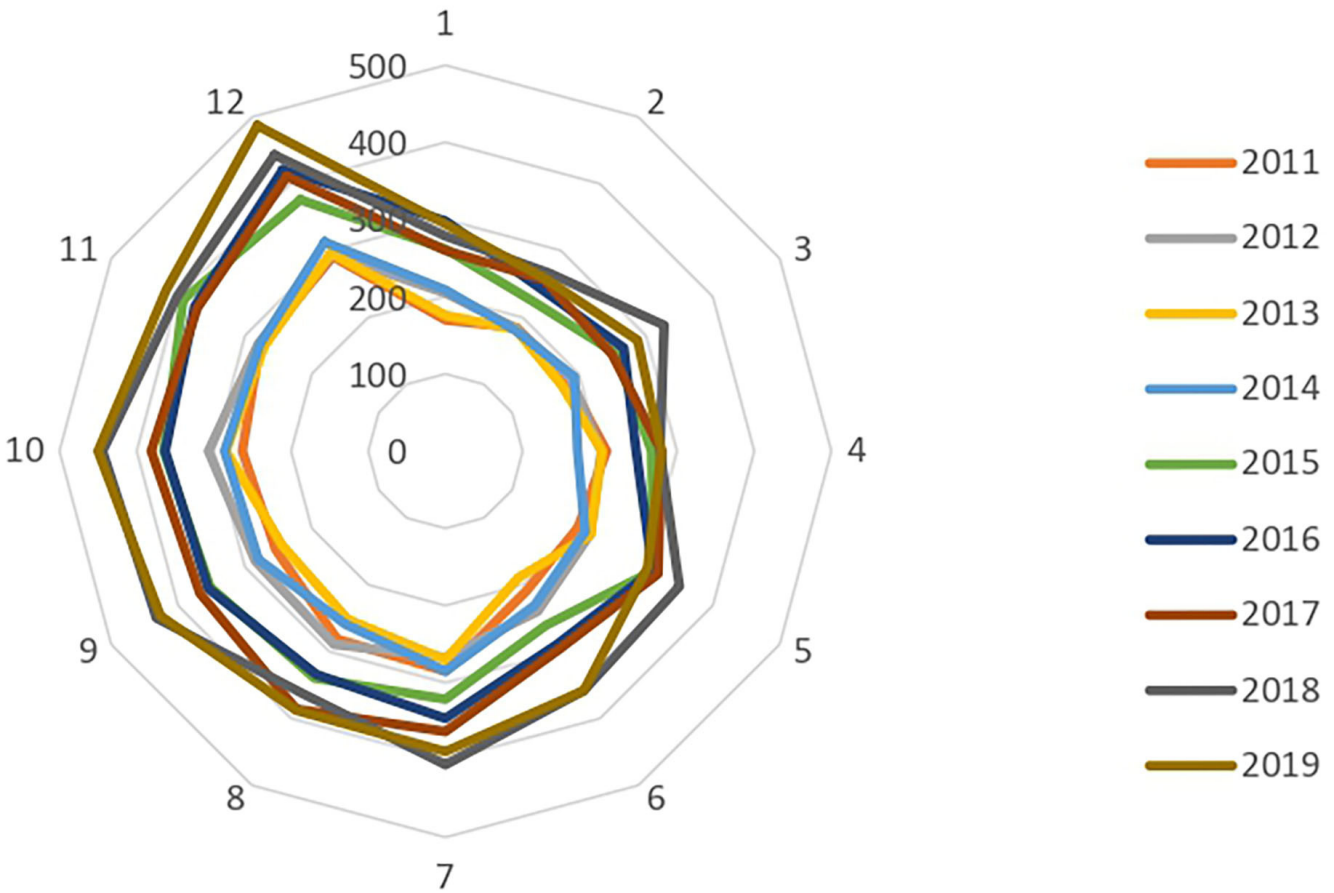
Birth months of children with ADHD at initial inpatient diagnosis between 2011 and 2019.

Thus, in 2011, children with an ADHD diagnosis born in December (291) were significantly more numerous than those born in January (170). This observation was confirmed throughout the period studied, since in 2019, among the 4,337 children coded as having ADHD as a principal diagnosis in the PMSI MCO and PSY databases, 487 were born in December and 294 in January. Overall, children born in December were 55% more likely to be diagnosed with ADHD than their peers born in January (minimum 41%, maximum 71% over the 2011–2019 period) (Figure 4).

#### Influence of the school system on psychostimulant prescription

**Figure 5:**
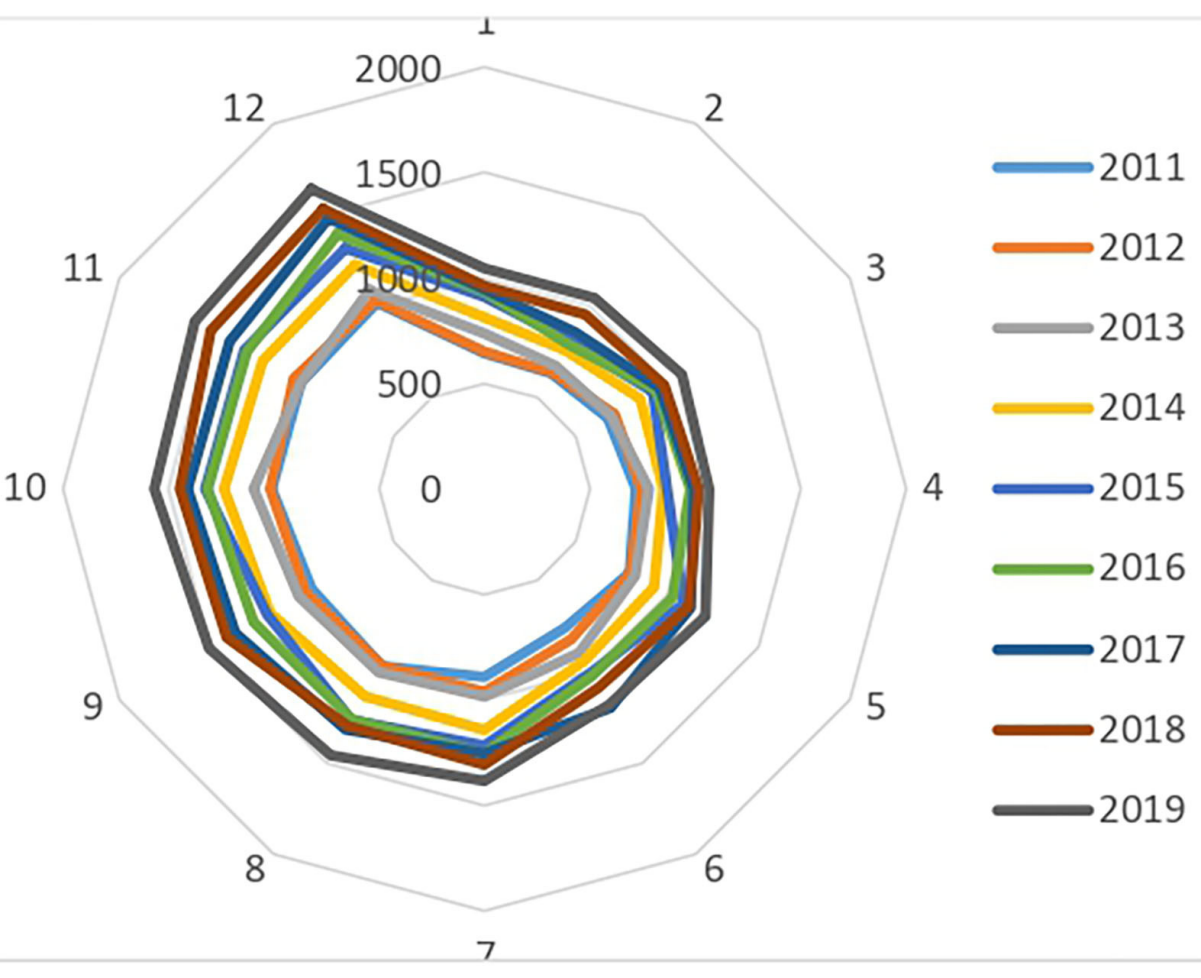
Children and adolescents treated with MPH by birth month between 2011 and 2019.

French children and adolescents are 44 to 60% more likely to be prescribed a psychostimulant treatment if they were born in December than if they were born in January (54% on average, over the entire period). In fact, the number of initial prescriptions increased progressively each year from January to December between 2010 and 2019, only to fall sharply in January of the following year (Figure 5).

#### ADHD: A socially determined diagnosis

Between 2010 and 2019, 35.4–38.8% of children diagnosed with ADHD lived in families receiving CMU or CMU-C (attributed to 7.8% of the French population). An ADHD diagnosis is therefore much more frequent among children from the most disadvantaged families. If we also consider children with ADHD diagnosed at hospital as being socially disadvantaged, the percentage of children with social difficulties in the ADHD cohort varies between 39.8 and 42.6% over the period (Table 8).

**Table 8:** Levels of social disadvantage among ADHD children and adolescents.

**Year**	**CMU + CMU-C**	**%**	**CMU + social disadvantage**	**%**	**Total population**
2010	1,217	38.3	1,305	41	3,181
2011	1,388	38.5	1,501	41.7	3,602
2012	1,591	38.8	1,734	42.3	4,100
2013	1,400	37	1,527	40.4	3,780
2014	1,520	38.5	1,667	42.2	3,950
2015	1,765	35.6	1,971	39.8	4,956
2016	1,944	36.2	2,211	41.2	5,373
2017	1,845	35.2	2,096	40	5,243
2018	2,200	36.9	2,538	42.6	5,957
2019	2,211	35.4	2,580	41.4	6,238

#### Social risk factors for MPH prescription

In 2019, 21.7% of children receiving MPH lived in families receiving CMU or CMU-C. This rate was much higher than the allocation of these aids in the general population (7.8%), and this trend increased between 2010 and 2019. If we also consider children taking MPH with a diagnosis of social disadvantages, the percentage of children with social difficulties among children taking MPH reaches 25.7% (Table 9).

**Table 9:** Levels of social disadvantage among children and adolescents using methylphenidate.

**Year**	**CMU + CMU-C**	**%**	**CMU + social disadvantage**	**%**	**Total population**
2010	4,240	14.4	5,254	17.9	29,402
2011	4,869	14.9	6,008	18.3	32,762
2012	5,475	15.2	6,733	18.7	36,014
2013	5,918	15.1	7,326	18.7	39,212
2014	6,833	15.7	8,380	19.3	43,477
2015	7,762	16.1	9,455	19.6	48,206
2016	8,705	16.6	11,088	21.1	52,574
2017	10,512	18.5	12,983	22.9	56,778
2018	12,495	20.6	15,017	24.7	60,762
2019	14,181	21.7	16,782	25.7	65,395

## Discussion

### What does the analysis of health databases tell us about the diagnosis and prevalence of ADHD in France?

Although the data available concerning the ADHD cohort do not allow establishing a reliable diagnostic or prevalence rate for France, the information dedicated to methylphenidate consumption in the population of child patients leads to adopt a critical stance concerning the prevalence information currently available. Indeed, between 2008 and 2011, the pharmaceutical industry financed a study concluding that there was a high prevalence of ADHD in France—between 3.5 and 5.6% in 2008 (48). This prevalence study was based on a telephone survey entrusted to non-specialist operators, trained on the fly, of a relatively small sample of the population: 1,012 households out of 7,012 randomly selected telephone numbers in the directory. According to these methodological criteria, the researchers estimated that 36 children (3.5%) aged between 6 and 12 suffered from hyperactivity/ADHD, while 22 children (2.2%) were treated with psychostimulants without being formally diagnosed [(48), p. 517]. The authors conclude that the prevalence rate of ADHD in France is between 3.5 and 5.6%. The study also shows that among the 3.5% of children diagnosed as hyperactive, 36.5% are also treated with MPH. Consequently, the study points to an MPH prescription rate of 3.48% among children aged 6–12 [2.2% + (3.5% × 0.365) = 3.48%].

Yet, a comparison of these data with MPH consumption rates listed in health databases shows clear exaggerations of the original study. In 2019, the prevalence of the consumption of MPH by children in France can be estimated at between 0.61 and 0.75% and has been steadily increasing since 2010. Previous publications, as well as the ANSM study published in 2017, estimated the prevalence of MPH use in 2008 (when the ADHD prevalence study was conducted) at 0.2% of children (49–54). The analysis of health databases, therefore, shows an overestimation of the results of the telephone survey supported by the drug industry in the face of the actual practices and prescriptions delivered by doctors in the country.

Moreover, the telephone survey did not take into account the complexity of psychological and diagnostic situations specific to child psychiatry. It did not include situations of co-diagnosis and co-morbidities: indeed, 50.6% of children hospitalized with an ADHD diagnosis between 2010 and 2019 had also received at least one other psychiatric diagnosis. Furthermore, one-third of the children in the MPH cohort had received a psychiatric diagnosis other than ADHD. The present study also supports the influence of the school and social system on ADHD diagnosis.

Previous international studies already reported such critical stance about ADHD prevalence estimations: serious studies have shown that the diagnosis of hyperactivity initially made in specialized centers was refuted in 62 to 78% of cases after re-evaluation (12, 14). Several subsequent studies have suggested that doctors or psychologists do not properly follow assessment procedures or comprehensive and clinical approaches meant to guide, confirm, or refute the diagnosis (15, 20). Furthermore, recurrent changes and the constant expansion of diagnostic criteria, as well as the emergence of subtypes, contribute to an exponential increase in prevalence rates and consequently to the increase in false-positives (11, 13, 21, 22).

### ADHD medication and MPH consumption: Comparison with existing data for France

On the one hand, among hospitalized children diagnosed with ADHD in 2018, 51.4% used MPH and 62% used one or more psychotropic drugs, most of which did not respect the recommendations of the French marketing authorization (*Autorisation de mise sur le marché*, or AMM). The duration of MPH treatment for these hospitalized ADHD children is particularly long (with a median of 7.1 years for children aged 6 in 2011). These long prescription durations and the massive use of co-prescriptions suggest that these children never get off the drug treatment and that MPH may be a gateway to medications that are not authorized for children in France.

On the other hand, consumption of MPH by children in France steadily increased between 2010 and 2019 in terms of incidence (+56.7%) and even more so in prevalence (+116%). A similar increase had already been reported for the period 2003–2005 (+65% prevalence) (52) as well as for the period 2005–2011 (+135%) (51). The report published by the ANSM in 2017 shows that between 2008 and 2014, the prevalence of MPH prescription for children aged 6–11 increased by 63% and almost doubled for adolescents aged 12–17 (49). Therefore, as in the previous studies, we show that the increase in MPH prescriptions continued at a consistently high rate from 2003 to the end of our study in 2019. As a result, previous, yet relatively recent, publications proposed prevalence estimates of MPH prescription to children that are much lower than those in 2019. These prevalence estimates vary: 0.14% in 2005 (50), 0.18% in 2005 (52), 0.2% in 2010 (53), and 0.25% in 2011 (51).^14^ According to our data, the prevalence of MHP prescription among 3–17-year-olds in 2019 would be between 0.61 and 0.75% of the general population.

Two previous studies presented data regarding the duration of MPH prescription (52, 54). The first reported that 16.6% of patients received only one prescription of MPH. The study also found that 33.8% of patients had short-term prescriptions (50% of which were interrupted during the first 3 months); for 49.6% of the patients, prescription renewal lasted longer (after 30 months, only 30% of prescriptions were interrupted). According to this study, the median duration of prescription in 2005 was 10.2 months (52). A more recent study carried out in the period 2010–2013 reported median treatment durations that were more consistent and longer the younger the patient [578 days for children under 6 years of age, 478 days for 6–11-year-olds and 303 days for 12–17-year-olds (54)]. Our observations are in line with this, since we observe the longest median durations in the youngest children. However, for 2011 we observe significantly longer median treatment times (between 1,990 days for 6-year-olds and 757 days for 11-year-olds). The difference between our observations and those of Pauly et al. (54) can be explained by the fact that the end date of treatment is not defined in the same way. In our study, this date is the date of the last prescription regardless of the duration of any interruptions in treatment, whereas for Pauly et al. (54), when treatment was interrupted for at least 90 days, this interruption date is taken into account.

Our study shows that MPH prescription does not always comply with the recommendations of the AMM and the regulations.

- Contrary to the recommendations of the AMM, MPH is sometimes prescribed before the age of 6. Although these prescriptions concern a limited number of children (4,390 in total between 2010 and 2019), they involve particularly long treatment times. The prescription to children younger than 6 years has been previously reported (50–54).
- MPH prescription in France is not necessarily associated with an ADHD diagnosis, which is the only authorized psychiatric indication for this drug. Moreover, when a psychiatric diagnosis is made, it does not always correspond to the therapeutic indication defined by the AMM. Indeed, the summary of product characteristics states that “psychostimulants are not intended for [...] patients with other primary psychiatric pathologies [...]”^15^.
- Contrary to the regulation in force until 13 September 2021, 25% of initial prescriptions and 50% of annual renewals are not made by a hospital specialist. Knellwolf et al. (52) had already noted that in 2003–2005, one-third of initial prescriptions were made outside the hospital. The same observation was also reported for the period 2010–2013 (54).
- Medical follow-up in the hospital services which initiated the treatment is not systematic (only 15.8–12.9% between 2011 and 2018). Consultations in CMPPs decline as the use of MPH increases, suggesting a progressive abandonment of the psychosocial treatment of ADHD. This treatment is nevertheless recommended as first-line treatment by the HAS.

In 22.8% of children and adolescents, we observed that MPH was prescribed in combination with at least one other psychotropic medication. A previous study had already reported relatively high rates (28.8%) of multiple prescriptions (50). According to Pauly et al. (54), 8% of 6–17-year-olds receiving a prescription for MPH were also prescribed an antipsychotic (mainly risperidone) while others (6%) received an anxiolytic in addition to MPH. Our observations thus confirm the practice of multiple prescriptions.

### ADHD medication and MPH consumption: Comparisons with other countries

French and international studies, even recent ones, have supported the idea that the prevalence of the prescription of MPH to children in France was particularly low compared to other European countries. This view was presented in a recent review comparing the prevalence of MPH prescriptions in 13 countries (24). Our data show otherwise. In Italy, the prevalence of MPH prescription to children in 2011 was only 0.17% (59), thus significantly lower than in France at the same period. In Great Britain, this prevalence has been stable since 2007 and is around 0.47% for children under 16 (60). Therefore, the prevalence in France as of 2019 is now higher than in Great Britain. In Denmark and Germany, prevalence has stabilized since 2009 at a level that is still higher than in France (1.5 and 2.2%, respectively) (23). However, these levels of prevalence could be quickly reached in France, as prevalence continues to increase rapidly. Other countries such as Holland, Iceland, and the United States have prevalence levels above 3%, but these levels have tended to stabilize since the end of the 2000s (23, 24, 59).

Our study and that of Pauly et al. (54) show that the duration of MPH prescription is longer the earlier the initial prescription is made. This trend has also been reported in Great Britain: 50% of children who started treatment between 6 and 10 years of age are still under treatment 4 years later, whereas the duration is < 2 years in 11–15-year-olds (61). Overall, the duration of MPH treatment appears to be quite similar in France and Great Britain both qualitatively and quantitatively. We did not find recent data for the other countries.

Other characteristics of MPH prescription in France have also been observed in many countries.

- First, MPH prescription concerns mainly boys (24). For example, in Great Britain, 85% of patients younger than 16 years are boys (61).
- Second, our study points to poly-prescriptions and a systematic undermining of the marketing authorization and recommendations of French health authorities, concerning both the treatment of ADHD and the prescription of MPH. Such multiple psychotropic prescriptions for ADHD and co-prescriptions of other psychotropic medications in combination with MPH have already been observed at the international level, particularly in the United States (62). Among the population of US children and adolescents prescribed at least one antipsychotic drug, the most frequent co-prescription was a psychostimulant and the most frequently associated diagnosis was ADHD, although it is not a recommended indication in the United States for the prescription of an antipsychotic drug (63). In the United States, the prescription of antipsychotics for children and adolescents increased significantly from the mid-1990s to the mid-2000s. However, in 2008, the American health insurance that manages the most disadvantaged families (Medicaid) became concerned about the serious side effects of antipsychotics (obesity, diabetes and drowsiness) which are even more severe than in adults (60) and controlled their prescription more strictly. Since this change in regulation, the prescription of antipsychotics to children has substantially decreased, in particular, for children younger than 8 years (64).
- Third, we show for the first time in France that school pressure contributes to the prevalence of MPH prescriptions. Indeed, children born in December receive this prescription more frequently than those born in January of the same year. This effect of schooling had already been observed in 13 countries (36).
- Fourth, our data show that MPH prescription is more common among children from the most disadvantaged families and that this trend increased between 2010 and 2019. Similarly, US children whose families are insured by Medicaid are more often prescribed a psychostimulant than those whose more affluent families are insured by private companies, and this gap increased between 2001 and 2010 (24).

### Accounting for the increase in MPH prescription in France: Social evolution, media bias, conflicts of interest, and the modification of care practices

MPH in France obtained its AMM in 1995. Between 1995 and 2001–2002, the prescription rate was very low (49). Since then, it has continued to increase at a much faster rate, as shown by both the data provided by the ANSM until 2014 (49) and the present observations concerning the period between 2010 and 2019. In the United States, Great Britain, and other European countries, the increase took place earlier, with a marked acceleration during the 1990s (23, 24, 61). However, in all these countries, the prescription of MPH to children either stabilized in the late 2000s or is clearly slowing down (23, 24, 61). One might therefore argue that the continuous increase we observe between 2010 and 2019 corresponds to France catching up with practices in comparable countries. However, according to this hypothesis, we should already be observing a slowdown in the increase in prevalence in the most recent years (2017–2019). This is not the case. Moreover, the recent change in regulations on 13 September 2021, which authorizes the initial prescription of MPH in out-of-hospital medical practice, will most likely further accelerate this increase.

The comparison between our ADHD hospitalized children and MPH cohorts suggests that the increase in MPH prescription in France is the result of the greater frequency of ADHD diagnosis. This may be due either to better recognition of ADHD or to a true increase in the frequency of ADHD in children. This second hypothesis is often rejected by proponents of a biological and a neuro-essentialist view of ADHD (65), but several factors such as school demands (36) or the rise of social inequalities (66) may have contributed to an increase in the frequency of ADHD symptoms in the last 20 years. In particular, excessive exposure to screens during childhood is a risk factor for ADHD (45–47), and this exposure increased significantly between 1997 and 2014, especially for children from age 0 to 2 years (from 80 to 190 min in the United States) (67). As this excessive exposure to screens in childhood is associated with family difficulties (68), including excessive screen use by mothers (69), it is not easy to establish the specific causal link between this excessive use and ADHD risk. It could be a combination of factors related to excessive screen exposure: less physical activity, obesity, infrequent language interactions with adults, lack of sleep, and emotional deprivation (70).

The fact that ADHD diagnosis is becoming more and more frequent in France is also the result of a societal evolution partly driven by the mass media. Since the beginning of the 2000s, television programs and websites dedicated to ADHD have proliferated in France. The vast majority of them support an organic cause for ADHD and present the drug treatment in an excessively favorable light (71, 72). The print media have also helped to raise awareness of ADHD but have been more nuanced about the use of MPH (73).

The pharmaceutical industry also played a role by funding the only ADHD prevalence study available in France, concluding a high prevalence rate (between 3.5 and 5.6% in 2008) (48), even though the data provided in support of this conclusion are highly questionable and in contradiction with the French healthcare system data. This same pharmaceutical company also funded the ADHD-France association, which actively advocates a biomedical approach to ADHD, including the prescription of medication and the lifting of the obligation of the first prescription of MPH by a hospital practitioner.^16^

Finally, the increase in the prescription of MPH could also result from a change in practices. In France, psychotherapy treatment, and educative and social interventions are recommended as first-line treatment for ADHD. When MPH is prescribed, it remains desirable to combine it with psychosocial treatment. This recommendation is in line with the main clinical guidelines, which recommend a multimodal approach (74). Our data show, however, that children receiving MPH have less and less recourse to CMPPs. For children from underprivileged families, psycho-social treatment, therefore, tends to give way to MPH alone. This weakening of the CMPPs suggests that, for these children, the recommendation for first-line psychosocial treatment is less and less respected.

## Conclusion

Our study shows that, concerning ADHD medication and the prescription of MPH to children, it is no longer possible to point to a French specificity. The prevalence of MPH prescription in France is now much higher than in Italy and has surpassed that of Great Britain. If the current progression continues at the same pace, France will soon catch up with Germany and Denmark. Indeed, in all countries comparable to France, there has been a stabilization of MPH prescription since the end of the 2000s. However, there is a concern that the current progression in France may accelerate given the 2021 authorization of initial out-of-hospital prescriptions. A large number of placebo-controlled studies have shown that MPH can reduce ADHD symptoms in most children. However, these are all short-term studies, and there is no evidence that the beneficial effects last more than a few months (74, 75). If short-term side effects are minor, long-term effects remain largely unknown (74). In any case, the co-prescription of MPH and an antipsychotic exposes the child to serious side effects and should be avoided (60). Finally, MPH is by no means the miracle pill touted by television programs, particularly with regard to school failure. Major North American studies have shown that the prescription of psychostimulants does not improve the academic performance of children suffering from ADHD (28). For all these reasons, the length of time during which MPH is prescribed in France is worrying, especially for the youngest children. This is why we advocate for an approach by which MPH prescription, if deemed necessary, should always be combined with a psychosocial treatment and that its relevance should be regularly evaluated.

Our study highlights an academic and social influence on ADHD diagnoses and MPH prescription. There is a proven risk of children being diagnosed and medicated as a function of their age or social origins. These discriminations compound unbridled breaches of prescription regulations that are supposed to underpin the democratic pact of confidence between citizens and their health care system. Faced with this situation, neither the AMM, nor the recommendations and reminder letters from the ANSM, nor warnings from researchers or health professionals who for many years have been denouncing these abuses seem to have been heard (76).

What benefits can we expect from the increasing medication of children's behaviors and from a progressive deregulation of the prescription of psychostimulants or even psychotropic drugs to child patients? Can children, adolescents, and their families find better support? Can the help provided to patients and care practices be improved? Can the link between the population, practitioners, and health services be strengthened?

These questions challenge the regulatory capacities of the medical community, health agencies, and public authorities, but they also touch on societal choices: what practices and what model of care do we want for our children and the next generations? These are sensitive and complex issues that at the very least deserve to be seriously debated by scientists, political authorities, and citizens themselves. This is all the more crucial as France is characterized by a culture of psychanalytic and psychologic care, educational practices, and social intervention which have proved their worth in the clinic and constitute a specificity of French psychiatry and psychopathology (77). It is on the importance and implementation of these practices that practitioners, researchers, and public policies must now focus.

## Data availability statement

The original contributions presented in the study are included in the article/supplementary materials, further inquiries can be directed to the corresponding author.

## Ethics statement

The study protocol has been validated by the Scientific Expertise Committee for Research, Studies, and Evaluations in the field of Health (Comité d'Expertise Scientifique pour les Recherches, les Études et les Évaluations dans le domaine de la Santé), case SNDS TPS 1190830 validated on 29-12-2019. It has also been validated by the French National Commission for Information Technology and Civil Liberties (CNIL) and an agreement with the National Health Insurance Fund (CNAM).

## Author contributions

SP obtained the funding, designed the study, contributed to the analysis of the data, and wrote the article. BT designed the study and analyzed the data. Both authors commented on the manuscript and approved its final version.

## Funding

This study was conducted with the financial support of the EOVI MCD Mutual Foundation (AP-EOV-18-002), in partnership with the Fondation de l'Avenir.

## Conflict of interest

SP declares that his research is conducted in the absence of any commercial or financial relationships that could be construed as a potential conflict of interest. SP le CIRNEF and the université de Rouen Normandie are responsible for this research. BT as founder and CEO of Median Conseil, has worked with several clients tied to the healthcare or nutrition domains, namely: Grunenthal, Lundbeck, Octapharma, Kiowa, Baxter, Physidia, Nestlé, Alexion, Amgen, Ipsen, Mallinkrodt, Merck, Pharma Mar, Takeda, Thea, 3M, Urgo. None of the studies conducted for these clients dealt with child psychiatry or psychotropic medications. BT and Median Conseil were responsible for processing the data for this research.

## Publisher's note

All claims expressed in this article are solely those of the authors and do not necessarily represent those of their affiliated organizations, or those of the publisher, the editors and the reviewers. Any product that may be evaluated in this article, or claim that may be made by its manufacturer, is not guaranteed or endorsed by the publisher.

## Footnotes

^1^The results concerning MPH prescription were published in the French-language academic journal *Neuropsychiatrie de l'enfance et de l'adolescence* in 2022: https://www.em-consulte.com/article/1501479/la-prescription-de-methylphenidate-chez-l-enfant-e. These data have not yet been published in English.

^2^For further information on the interest and use of French health insurance databases, see Bezin et al. (57) and Moulis et al. (58).

^3^All situations of pathologies (psychiatric or other) are referenced in health databases according to the coding of the International Classification of Diseases (ICD-10).

^4^The ACS allows people with resources slightly higher than those of CMU beneficiaries to have access to mutual insurance coverage. The CMU, CMU-C, and ACS are schemes reserved for socially disadvantaged people or those with low incomes.

^5^Diagnoses of social disadvantages are listed in the ICD-10 nomenclature under the heading “Subjects whose health may be threatened by socioeconomic and psychosocial conditions.” We referred to the following codings: Z55 Difficulties related to education and literacy; Z56 Difficulties related to employment and unemployment; Z57 Occupational exposure to risk factors; Z58 Difficulties related to the physical environment; Z59 Difficulties related to housing and economic conditions; Z60 Difficulties related to the social environment; Z61 Difficulties related to an unhappy childhood; Z62 Other difficulties related to education; Z63 Other difficulties related to the immediate environment, including family situation; Z64 Difficulties related to certain psychosocial situations; Z65 Difficulties related to other psychosocial situations.

^6^https://www.health-data-hub.fr/cesrees (reference TPS 1190830).

^7^ https://www.cnil.fr/

^8^https://ansm.sante.fr/actualites/methylphenidate-donnees-dutilisation-et-de-securite-demploi-en-france; https://ansm.sante.fr/dossiers-thematiques/medicaments-en-pediatrie-enfants-et-adolescents/classes-dage-des-enfants-et-adolescents; https://www.ema.europa.eu/en/documents/scientific-guideline/international-conference-harmonisation-technical-requirements-registration-pharmaceuticals-human-use_en-1.pdf

^9^The reader can contact the first author for any additional methodological indication.

^10^This was based on the distribution of psychotropic drugs ATC N03, N04, N05, and N06 in the 12 months following initial ADHD diagnosis. Due to these methodological choices and the lack of hindsight, the results for the year 2019 cannot be taken into account.

^11^Recall that what we measure here is the rate of consumption of psychotropic drugs, taking into account all compounds, in the year following the patients' hospitalization. For this reason, the rate of psychostimulant medication for this section of the results is lower than in the section dedicated to MPH consumption, which is calculated for the entire study period. It is likely that the analysis of the consumption of psychotropic medication among ADHD patients hospitalized over the entire period covering 2010–2018 would have led to medication rates close to 100%.

^12^We refer here to the official INSEE count, updated in 2020. The data are available at: https://www.insee.fr/fr/statistiques/4277748?sommaire=4318291 (accessed June 13, 2021).

^13^Given the method of analysis (medical consultations over a 13-month period after the beginning of treatment), we did not include the data from 2019. The observable differences between the number of children receiving at least one medical follow-up in the year following the beginning of treatment and the rate of renewal of the initial hospital prescription at 13 months can be explained by the fact that in a number of cases, the hospital prescription was renewed by a different service from the one that initiated the treatment.

^14^One of the studies, conducted in the Provence Alpes Côte d'Azur region, gives an estimate of the prevalence in 2011, all ages combined, of 0.68‰ (51). It states that 75.3% of MPH prescriptions were for children and adolescents aged 17 or younger. If we leave out children under 3, for whom the prescription of MPH remains exceptional, and if we take into account the fact that the population of 3–17-year-olds represents about one fifth of the French population, we can deduce from this study a prevalence of about 0.25% for 3–17-year-olds in 2011.

^15^http://agence-prd.ansm.sante.fr/php/ecodex/rcp/R0141880.htm (accessed June 13, 2021).

^16^ https://www.tdah−france.fr/Fin−de−la−Prescription−Initiale−hospitaliere−PIH−pour−le−methylphenidate.html#:~:text=A%20partir%20du%2013%20septembre,en%20p%C3%A9diatrie%20ou%20en%20psychiatrie
